# What is the impact of birth weight corrected for gestational age on later onset asthma: a meta-analysis

**DOI:** 10.1186/s13223-021-00633-3

**Published:** 2022-01-04

**Authors:** Jingjing Wang, Zeyi Zhang, Ou Chen

**Affiliations:** School of Nursing and Rehabilitation, Shandong University, NO.44, Wenhua West road, Jinan, Shandong China

**Keywords:** Asthma, Birth weight, Gestational age, Small for gestational age, Large for gestational age

## Abstract

**Background:**

Asthma is a common multifactorial disease affecting millions worldwide. The Barker hypothesis postulates an association between later onset disease risk and energy exposure in utero. Birth weight corrected for gestational age is better for measuring the infant size, which reflects energy exposure in utero. Findings on asthma and birth weight corrected for gestational age have been inconclusive. We conducted a meta-analysis to further clarify the relationship between birth weight corrected for gestational age and later onset asthma.

**Methods:**

A systematic literature search of the PubMed, Web of Science, MEDLINE, and Scopus databases up to January 2021 was conducted. The subject terms were used as follows: “asthma”, “allerg*”, “respiratory”, “birth weight”, “gestational age”, “birth outcomes”, “intrauterine growth retardation”, and “fetal growth restriction”.

**Results:**

We included 12 articles with data from a total of 6,713,596 people. Compared with non-SGA infants, infants small for gestation age (SGA) were not associated with an increased risk of asthma (OR = 1.07; 95% CI 0.94–1.21). However, in the subgroup analysis, we found an increased risk of later onset asthma among SGA in studies conducted in Asia, with a large sample size, and defined asthma through medical records rather than questionnaires. Large for gestational age (LGA) was not associated with an increased risk of asthma when non-LGA or appropriated for gestational age (AGA) infants were used as the reference (OR = 1.02; 95% CI 0.90–1.16; OR = 1.01; 95% CI 0.88–1.15).

**Conclusion:**

These results indicated that neither SGA nor LGA was associated with an increased risk of asthma. However, considering the limitations of the research, these results should be interpreted with caution.

## Background

Asthma is a common multifactorial disease affecting millions worldwide [1]. Its etiology is increasingly attributed to interactions between genetic predisposition, host factors, and environmental exposures [2]. The evidence supports the hypothesis that environmental changes play a significant role in the current asthma epidemic. Environmental triggers may affect asthma differently during different times of a person’s life, and the relevant risk factors may change over time [3].

However, most studies assessing risk factors for asthma development have been limited to life events that occur long after birth. Humans are influenced by various environmental factors from the moment of conception [4]. The Barker hypothesis postulates that an important component of adult disease risk is determined in utero, with maternal nutrition playing an important role [5]. Size for gestational age at birth reflects energy exposure and transfer and the placental function during pregnancy [6]. Barker et al. also implicated an association between birth weight and later respiratory disease [5]. Subsequently, many studies have examined the relationship between asthma and birth weight, but the conclusions were inconsistent. Recently, a meta-analysis reported an increased risk of childhood asthma with low birth weight (LBW), while high birth weight (HBW) was not associated with an increased risk of asthma [7].

However, a major limitation of many of these studies was that they did not consider the gestational age of the infants. LBW may be a proxy for prematurity, which is an independent risk factor for respiratory morbidity in childhood [8]. Therefore, birth weight corrected for gestational age (BW/GA) is better for measuring infant size and predicting long-term health concerns. Being small for gestational age (SGA) or large for gestational age (LGA) has been associated with adverse neonatal and infant outcomes and developmental outcomes during childhood and beyond [9–11]. Some researchers have found that both SGA [12, 13] and LGA [14] are associated with an increased risk of later asthma. However, some researchers reported BW/GA had little effect on later asthma [15, 16]. Therefore, the objective of our meta-analysis was to estimate the direction and magnitude of the impact of BW/GA on later asthma.

## Methods

### Literature sources

We performed a literature retrieval including the databases PubMed, Web of Science, MEDLINE, and Scopus using the terms “asthma”, “allerg*”, “respiratory”, and “birth weight”, “gestational age”, “birth outcomes”, “intrauterine growth retardation”, and “fetal growth restriction” in the title. The search was conducted through January 2021.The references of the literature selected for the present study were also examined to improve the recall rate.

### Study inclusion and exclusion criteria

To be eligible, a study must meet the following criteria: it must have been an original document assessing the relationship between BW/GA and asthma, and there were sufficient data to calculate the required results. In addition, BW/GA should be reported in categories within accepted ranges (e.g., SGA, infants with a birth weight below the 10th percentile for GA; appropriate for gestational age (AGA), birth weight between the 10th and 90th percentile for GA; LGA, birth weight above the 90th percentile for GA). The reasons for exclusion were as follows: (1) if the paper was a review or comment; (2) if the full text could not be obtained; (3) it was a duplicate.

### Primary variables

The primary outcome was asthma and the diagnosis was obtained from medical records or questionnaires in all included studies. The main exposure was BW/GA, including SGA, AGA, LGA.

### Study selection

First, two reviewers screened the literature by reading titles and abstracts independently. Then, they read the full text to determine whether the previously selected articles fulfilled the inclusion criteria. They were included in the meta-analysis if they fulfilled the eligibility criteria. If there were discrepancies between the reviewers, the inclusion or exclusion of the article was decided by the third reviewer.

### Evaluation of study quality

The Newcastle–Ottawa instrument recommended by the Cochrane Collaboration was used to assess the quality of the included cohort and case–control studies. It contains eight questions in three areas: selection, comparability, outcome or exposure. The article receives a star when it meets one term criterion, and the criteria for the grades of study quality are (1) low quality—when the article gets no more than 3 stars, (2) moderate quality—gets 4 to 6 stars, and (3) high quality—gets 7 to 9 stars. The cross-sectional study appraisal tool developed by the Joanna Briggs Institute (JBI) was used to assess the cross-sectional studies and it contains eight evaluation items. Studies were assessed based on the subjects, diseases, the measurement of influencing and confounding factors and the data analysis. The reviewers use “yes”, “no”, “unclear” or “unsuitable” for judging each item.

### Data extraction

Data were extracted from the included studies by two researchers separately and then they were cross-checked. The data contained the following fields: author’s name, year of publication, country of origin, study design, characteristics of the participants, and the outcomes.

### Data analysis

Statistical analysis was conducted using STATA 14.0. The odds ratio (OR) was used as the measure for dichotomous outcomes, and the 95% confidence interval (CI) for each outcome was estimated to reflect the uncertainty of the point estimates. The unadjusted risk estimates were calculated only when the adjusted risk estimates were unavailable. The effect model for the statistical calculation was selected according to the heterogeneity. Statistical heterogeneity was assessed by chi-square test and it was combined with the I-squared (I^2^) value to quantitatively judge the heterogeneity. A fixed-effects model was applied when there was no significant heterogeneity (*I*^*2*^ ≤ 50%). Otherwise, the random-effects model was used (*I*^*2*^ > 50%). Subgroup analyses and sensitivity analysis were applied to explore the potential sources of heterogeneity, and the latter was also performed to assess the robustness of the results. Publication bias was evaluated by using Egger’s test [17].

## Results

### Selected studies

**Fig. 1:**
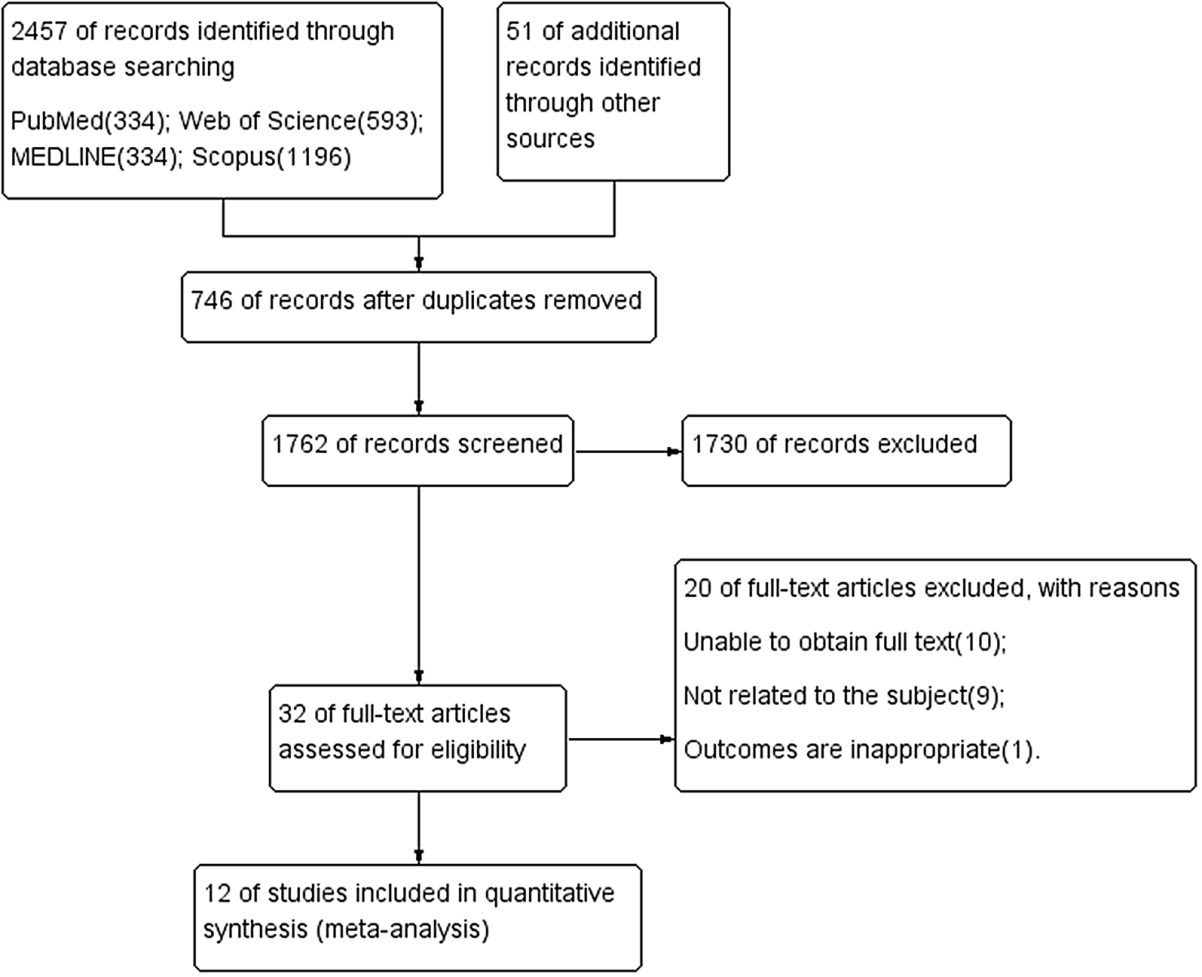
References searched and selection of articles in the meta-analysis

A total of 2508 articles were retrieved from the databases. After excluding duplicates and reading the titles and abstracts, 32 articles were selected for full-text reading. Only 12 articles with a total of 6,713,596 people met the inclusion criteria and were included in this meta-analysis (Fig. 1). The years of publication for the included studies were 2002 to 2019.

### Basic characteristics and the quality of the studies

Among the 12 articles, there were 6 cross-sectional studies, 5 cohort studies and 1 case–control study. Twelve articles reported fourteen original studies (Gessner [12] divided subjects into two groups according to age; Lu [18] counted two sets of data based on different ways of obtaining the diagnosis of asthma) and they were included in the meta-analysis. Five articles [13, 15, 16, 19, 20] provided adjusted risk estimates, and we used the ORs and 95% CIs to combine the effect sizes. Cohort and case–control articles were assessed as high-quality studies, while most of the cross-sectional papers did not describe the exclusion criteria or the demographic characteristics of the subjects (Tables 1 and 2). The general characteristics of the studies are shown in Table 3.

**Table 1:** Assessment of the case–control and cohort study quality

Studies	Selection				Comparability		Outcome/Exposure			Score
	1	2	3	4	5a	5b	6	7	8	
Hesselmar et al. [19]	*		*	*	*		*	*	*	******
Grischkan et al. [21]	*	*	*	*				*	*	******
Jaakkola and Gissler [16]	*	*	*	*	*		*	*	*	*******
Liu et al. [22]	*	*	*		*		*	*	*	*******
Pinto et al. [23]	*	*	*	*	*			*	*	*******
Carter et al. [13]	*	*	*	*	*		*	*	*	*******

For cohort studies, 1, representativeness of the exposure group; 2, representativeness of the non-exposed group 3, determination of exposure; 4, interesting outcome not present in the beginning; 5a, controlling the most important factor; 5b, controlling any factors; 6, determination of the outcome; 7, long follow up until the outcomes to appear; 8, integrity of the study follow-up

For case–control studies, 1, appropriate case identification; 2, cases are representative; 3, appropriate source of the control group; 4, no targeted medical history in the control group; 5a, confounding of the most important factors; 5b, confounding of any factors; 6, appropriate determination of the exposure factors; 7, determination that the exposure factors are the same in both groups; 8, no response rates

*The article meets this term criterion

**Table 2:** Assessment of the cross-sectional study quality

Studies	1	2	3	4	5	6	7	8
Gessner and Chimonas [12]	No	Yes	Yes	Yes	Yes	Yes	Yes	Yes
Lu et al. [18]	No	Yes	Yes	Yes	Yes	Yes	Yes	Yes
Wang et al. [20]	No	No	Yes	Yes	Yes	Yes	Yes	Yes
Kalen et al. [14]	No	No	Yes	Yes	Yes	Yes	Yes	Yes
Koshy et al. [24]	No	No	Yes	Yes	Yes	Yes	Yes	Yes
Miyake and Tanaka [15]	No	No	Yes	Yes	Yes	Yes	Yes	Yes

For cross-sectional studies, 1, inclusion criteria of subjects; 2, describe the study subjects and site; 3, assessment of exposure factors; 4, assessment of health problems; 5, clarification of confounding factors; 6, control confounding factors; 7, evaluation of outcome indicators; 8, appropriate data analysis methods

**Table 3:** Characteristics of the 12 studies included in the meta-analysis

Author, publication year	Country	Study design	Total number of subjects	Age	Asthma definition	BW/GA	Gestational age
Hesselmar et al. [19], 2002	Sweden	Case–control	950	15–25y	Questionnaire	SGA	–
Grischkan et al. [21], 2004	American	Cohort	251	8–11y	Questionnaire	SGA	24–36w
Jaakkola and Gissler [16], 2004	Finland	Cohort	58,841	0–7y	Medical record	SGA	–
Gessner and Chimonas [12], 2007	American	Cross-sectional	37,349	< 10y	Medical record	SGA	–
Lu et al. [18], 2012	Taiwan	Cross-sectional	75,181	10–17y	Medical record and Questionnaire	SGA; AGA; LGA	–
Wang et al. [20], 2012	Taiwan	Cross-sectional	78,011	13–16y	Questionnaire	SGA	–
Kalen et al. [14], 2013	Sweden	Cross-sectional	763,666	2–11y	Medical record	SGA; AGA; LGA	23–44w
Koshy et al. [24], 2013	UK	Cross-sectional	6361	5–11y	Questionnaire	SGA	39–41w
Miyake and Tanaka [15], 2013	Japanese	Cross-sectional	2004	3y	Questionnaire	SGA	–
Liu et al. [22], 2014	Sweden Finland Denmark	Cohort	5,656,507	3–18y	Medical record	SGA; LGA; AGA	22–45w
Pinto et al. [23], 2017	Netherlands	Cohort	1608	8y	Questionnaire	LGA	38–42w
Carter et al. [13], 2019	Canada	Cohort	32,867	0–25y	Medical record	SGA; LGA; AGA	≥ 37w

*BW/GA* birth weight corrected for gestational age, *SGA* small for gestational age, *AGA* appropriate for gestational age, *LGA* large for gestational age, *y* year, *w* week

### BW/GA and risk of asthma

#### SGA and asthma

**Fig. 2:**
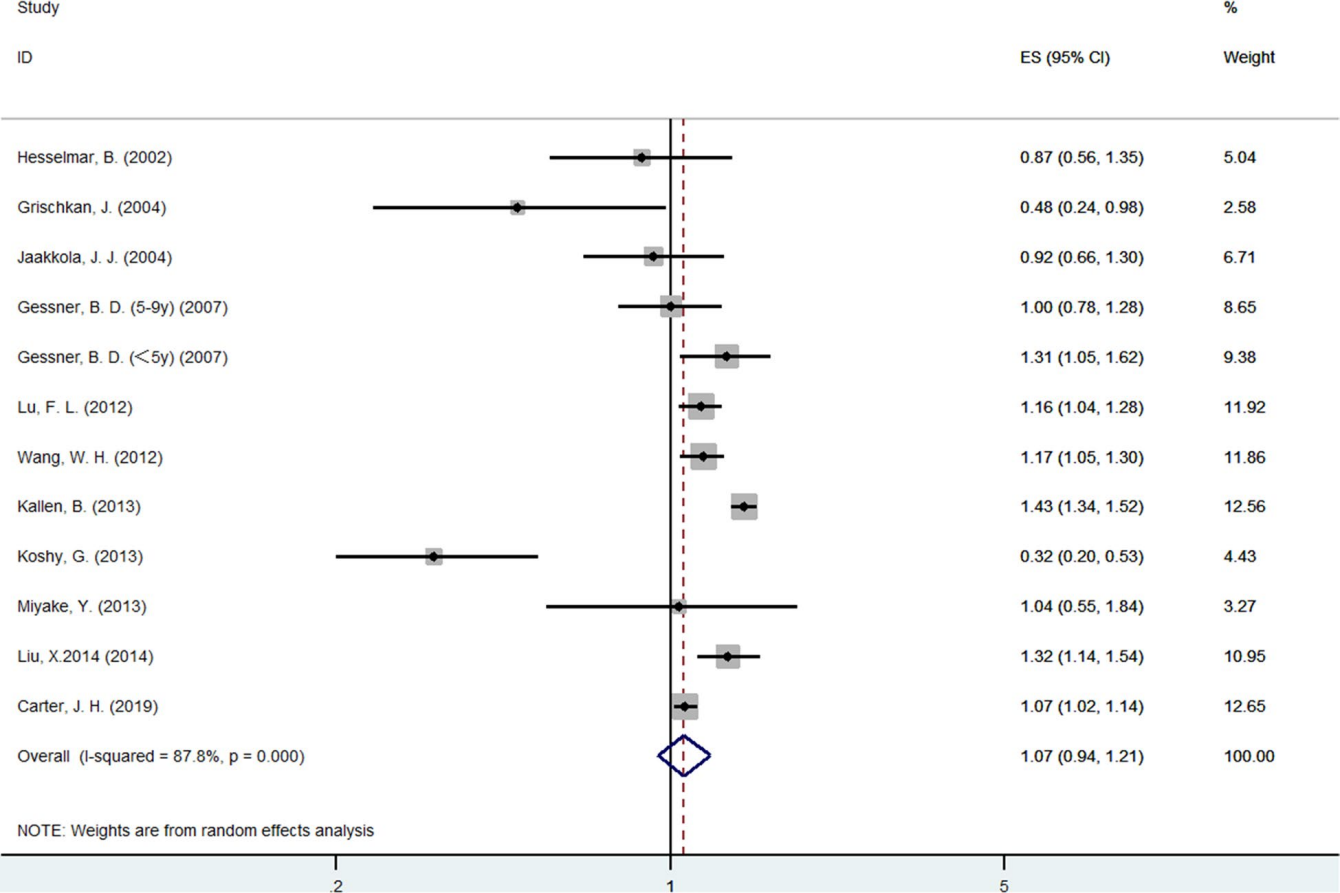
A meta-analysis of asthma prevalence in subjects with SGA compared with non-SGA. Horizontal lines indicate 95% CI, and the pooled OR was analyzed by using a random-effects model

Twelve studies [12–16, 18–22, 24] provided data on the asthma prevalence in subjects with SGA compared with non-SGA subjects. We conducted a cumulative meta-analysis according to the publication year. Data from these studies were pooled using the random-effects model (*I*^*2*^ = 88%, *P* = 0.032), and the results from this analysis revealed that infants with SGA were not associated with an increased risk of later onset asthma (OR = 1.07; 95% CI 0.94–1.21) (Fig. 2).

Seven studies [12, 14–16, 21, 24] reported the effects of SGA on childhood asthma (under the age of 14), and our pooled result (OR = 0.90; 95% CI 0.66–1.22) showed that SGA did not increase the risk of childhood asthma when compared with non-SGA. We also found that full-term (GA > 36 weeks) infants with SGA [13, 14, 22, 24] were not associated with an increased risk of later onset asthma (OR = 0.98; 95% CI 0.79–1.23) (Table 4).

**Table 4:** Results of the meta-analysis

Meta-analysis	Number of studies	*I*^*2*^	Model	OR (95% CI)
SGA vs. non-SGA (< 14y)	7	89.30%	Random	0.90 (0.66–1.22)
SGA vs. non-SGA (GA > 36w)	4	91%	Random	0.98 (0.79–1.23)
SGA vs. AGA	5	95%	Random	1.08 (0.89–1.32)
LGA vs. non-LGA	5	89%	Random	1.02 (0.90–1.16)
LGA vs. AGA	4	92%	Random	1.01 (0.88–1.15)

*SGA* small for gestational age, *AGA* appropriate for gestational age, *LGA* large for gestational age, *y* year, *w* week

Table 4 also shows the impact of SGA on later onset asthma compared with AGA infants [13, 14, 18, 22, 24]. A random-effects model was used to calculate the effect size, and the results revealed that infants with SGA did not have an increased later asthma risk (OR = 1.08; 95% CI 0.89–1.32).

#### LGA and asthma

Non-LGA [13, 14, 18, 22, 23] and AGA [13, 14, 18, 22] were used as the reference categories to assess the increased risk of asthma in subjects with LGA. The random-effects model was used to calculate the effect size (*I*^*2*^ = 89%, *P* = 0.015; *I*^*2*^ = 92%, *P* = 0.015), and the pooled estimates revealed no association between LGA and an increased risk of later onset asthma (OR = 1.02, 95% CI 0.90–1.16; OR = 1.01, 95% CI 0.88–1.15) (Table 4).

### Subgroup analyses

Subgroup analyses of SGA compared with non-SGA were stratified by study design, study sites, sample size, and methods of obtaining the diagnosis of asthma to investigate the potential sources of heterogeneity. The results of the subgroup analyses revealed that SGA was associated with an increased risk of asthma in studies conducted in Asia, with sample sizes ranging from 50,000 to 100,000 and more than 100,000, and they defined asthma through medical records. The sample size could probably be regarded as the source of the heterogeneity between studies (Table 5).

**Table 5:** SGA vs. non-SGA: the subgroup analysis

Stratification		Number of studies	*I*^*2*^	Model	OR (95% CI)
Overall		12	87.80%	Random	1.07 (0.94–1.21)
Study design	Case–control	1	–	Random	0.87 (0.56–1.35)
	Cohort	5	76.60%	Random	1.05 (0.86–1.29)
	Cross-sectional	6	88.80%	Random	1.08 (0.91–1.29)
Study site	Europe	5	91.20%	Random	0.94 (0.69–1.28)
	North America	4	65.10%	Random	1.06 (0.88–1.27)
	Asia	3	0%	Random	1.16 (1.08–1.25)*
Sample size	< 5000	3	30.7%	Random	0.80 (0.54–1.18)
	5000–10,000	1	–	Random	0.32 (0.20–0.52)*
	10,000–50,000	3	43.20%	Random	1.10 (0.98–1.25)
	50,000–100,000	3	0%	Random	1.15 (1.07–1.24)*
	> 100,000	2	0%	Random	1.41 (1.33–1.50)*
Asthma definition	Questionnaire	5	87.50%	Random	0.72 (0.43–1.20)
	Medical record	7	88.70%	Random	1.19 (1.04–1.35)*

*SGA* small for gestational age

*P < 0.05

### Sensitive analysis

Sensitive analyses were conducted for the above six outcomes. We found that the results were robust, except for the outcome of the risk of asthma in subjects with SGA compared with those with non-SGA when the gestational age was greater than 36 weeks. Koshy, G. may be the source of heterogeneity in this outcome.

### Publication bias

Examination of the included investigations did not show a significant effect of publication bias. We assessed the potential publication bias by applying Egger’s test, and it was not statistically significant (*P* = 0.15).

## Discussion

In the present meta-analysis, 12 articles reporting 14 original studies with 6,713,596 subjects were included. By conducting a systematic review, we estimated that there was no association between BW/GA and an increased risk of later asthma. After removing the studies of Grischkan [21], Koshy [24], and Miyake [15], the cumulative meta-analysis showed that the estimate gradually became consistent, revealing that SGA was associated with an increased risk of asthma when non-SGA was used as the reference, and the corresponding CIs narrowed down in the order of publication year. In the subgroup analyses, the effects of SGA on asthma were statistically significant in Asia, those with a large sample size, and those defined asthma through medical records.

SGA is defined as infants with a birth weight below the 10th percentile for GA or below 2 S.D. of the reference population mean for BW/GA, which is different from the preterm or LBW population [25]. SGA infants are divided into two categories: SGA infants with normal constitution and SGA infants who have a birth weight lower than the expected optimal birth weight because of growth restriction [26]. The former has a normal birth weight less than the 10th percentile because of inherent factors such as maternal height, weight, ethnicity, and parity, and among these infants, there is no increased risk of perinatal mortality or morbidity, while the latter has a higher risk of mortality and morbidity during the neonatal period and beyond [27]. There are many possible reasons to explain the later result. Children born with fetal growth restriction (FGR) have a greater risk of developing bronchopulmonary dysplasia [28], which is associated with childhood asthma [29], providing a potential mechanism by which SGA increases the risk of asthma [22]. Moreover, factors leading to FGR may also cause “programming” of the respiratory or immune system [30], predisposing SGA infants to develop asthma. Most of the articles we included did not specify which group of SGA was used, perhaps misclassification makes the result meaningless.

Some studies found that SGA was strongly associated with an increased risk of asthma only when subjects were stratified by certain factors, such as maternal smoking^16^, lower respiratory infection [12], and childhood overweight [18]. These results are consistent with the theory of perinatal synergistic mechanisms [16, 31, 32], which suggests that perinatal factors alone have little or no effect on the development of asthma or confer only a slight increase in risk. But if there are associated risk factors, such as prematurity plus maternal smoking during pregnancy, the risk for asthma is notably increased. However, the number of such articles was so low that we could not carry out a meta-analysis.

Subgroup analysis for the sample size showed low heterogeneity among the subgroups, and SGA became a risk factor for asthma when the sample size was more than 50,000. A large sample size is more representative, and a more accurate estimation can be obtained to improve the accuracy of the results. In the forest plot, the 95% CIs for large samples were narrower. A twin study showed that low birth weight is associated with adult-onset asthma, and the analysis suggested that the findings were unlikely to be confounded by genetic or shared environmental factors [33]. Therefore, the association between SGA and an increased risk of later asthma may be influenced by the asthma phenotype. The composition of the asthma phenotype varies in different regions, thus subgroup analysis according to region produced inconsistent results. Further research can focus on the phenotype to explore the relationship between BW/GA and asthma. In the sensitivity analysis of SGA versus non-SGA (GA > 36 weeks), we found that Koshy [24] may be the source of heterogeneity, which estimates SGA as a protective factor for asthma. Compared with the other three articles, this may be because only children between 39 and 41 weeks of gestational age were included, and the asthma definition used a questionnaire.

Studies on the relationship between LGA and asthma were contradictory; some articles found no correlation [22, 23], while some reported a positive correlation [14] and suggested LGA infants have a higher risk of later obesity, which is an obvious risk factor for asthma. In the present meta-analysis we found that LGA was not associated with an increased risk of asthma when infants with non-LGA or AGA were used as reference. This conclusion is consistent with a previous systematic review on the relationship between HBW and asthma. The possible reasons for the meaningless results are as follows [7]. Infants with LGA are associated with an increased risk of overweight in childhood and adulthood [34–36]. Overweight is an independent risk factor for asthma [37, 38]. However, LGA may have no direct effect on later asthma after controlling for confounders. Unfortunately, the studies included in our research did not distinguish the effect of LGA on childhood asthma and adult asthma. These results should be prudently treated because only a small number of studies were included.

Several limitations of this meta-analysis should be considered. First, half of the included articles were cross-sectional, which could not provide a direct causal link between BW/GA and asthma. However, most of these studies had a large sample size, and the findings in cross-sectional studies were consistent with those in cohorts. Second, many studies did not provide a specific gestational age, which may be a potential source of bias in the present article. Preterm birth is associated with LBW, which is a risk factor for asthma. However, we found that the outcomes in full-term infants with SGA were consistent with those in infants unrestricted gestational months. Furthermore, the diagnosis of asthma in young children is considered less accurate because of its clinical instability in the early years of life [22]. Last, the effect of BW/GA on asthma was not the primary objective of most of the identified studies, possibly leading to missing relevant data that were not evident in the title or abstract. These limitations must be noted, and the results should be considered with caution.

## Conclusion

Generally, this meta-analysis showed that infants with SGA or LGA were not associated with an increased risk of later asthma. Additional stratified analyses need to be published to explore the effect of perinatal synergistic mechanisms on asthma.

## Data Availability

The datasets used and analyzed during the current study can available from the corresponding author on reasonable request.

## Abbreviations

LBW: Low birth weight
HBW: High birth weight
SGA: Small for gestational age
LGA: Large for gestational age
BW/GA: Birth weight for gestational age
AGA: Appropriate for gestational age
OR: Odds ratio
CI: Confidence intervals
